# Ectopic Endometrial Cell-Derived Exosomal Moesin Induces Eutopic Endometrial Cell Migration, Enhances Angiogenesis and Cytosolic Inflammation in Lesions Contributes to Endometriosis Progression

**DOI:** 10.3389/fcell.2022.824075

**Published:** 2022-04-26

**Authors:** Maidinaimu Abudula, Xiaodan Fan, Jing Zhang, Jiajie Li, Xiaoming Zhou, Yichen Chen

**Affiliations:** ^1^ Department of Gynecology, Ningbo Women and Children’s Hospital, Ningbo, China; ^2^ Medical School, Ningbo University, Ningbo, China; ^3^ Department of Gynecology, The Affiliated Hospital of Medical School of Ningbo University, Ningbo University, Ningbo, China; ^4^ Department of Pharmacology, Ningbo Institute of Medical Science, Ningbo, China

**Keywords:** exosomal moesin, endometriosis, migration, vascularization, inflammation

## Abstract

**Background:** Endometriosis (EMs) is the most common gynaecological disorder with its etiology and/or pathophysiology remains enigmatic. Recent studies showed that extracellular vesicles (EVs), exosomes in particular, play a critical role in developing various clinical disorders. However, the implication of exosomes in endometriosis progression has not been well elucidated.

**Method:** The ectopic stromal cellular exosomes (eEVs) were assessed by transwell assay, scratch tests, tube formation assay, western blot, and qRT-PCR analysis. Protein expression profiles of exosomes in endometrial tissue and vaginal discharge collected from patients with EMS and healthy donors were analysed by Mass spectrometry. siRNA interference technology was used to inhibit the expression of exosomal protein for the functional analysis in *in-vivo*. Finally, *in-vitro* experiments were performed to validate the results that we observed in EMs mouse model.

**Results:** *In vitro*, we discovered that eEVs improved NSC migratory potential by upregulating MMP9 expression and activity. eEVs also aided angiogenesis and elevated the expression of inflammatory cytokines in ovarian epithelial cells, according to our findings. Moesin (MSN) levels in ESC exosomes were substantially greater than in NSC exosomes (1.22e^8^±5.58e^6^ vs. 6.605e^7^±4.574e^6^, LFQ intensity), as shown by protein mass spectrometry and bioinformatics analysis. In ectopic stromal cells, ERa receptors stimulated the RhoA/Rock-2/MSN pathway. We discovered that downregulating exosomal moesin reduced NSC migration (about 3-fold change) and MMP9 expression (about 2-fold change). On the other hand, Exomsni inhibited angiogenesis and inflammatory cytokine release. *In vivo* the result of immunohistochemistry and immunofluorescence demonstrated that exosomal MSN substantially modified the expression of MM9, VEGFR and p-VEGFR in polyclonal lesions. In addition, we discovered an elevation in the expression of proinflammatory factors in the surrounding tissue.

**Conclusion:** Exosomal MSN derived from ectopic stromal cells can contribute to endometriosis progression by mediating the construction of a “migration-vascularization-inflammation” loop in the ectopic environment.

## Introduction

Endometriosis (EMs) is a chronic and inflammatory gynecological condition in which the implantation and growth of endometrial tissue outside the uterus cavity (Miller et al., 2017). EMS affects up to 10% of women of reproductive age, accompanied by chronic pelvic pain, irregular menstruation, and infertility in as many 50% of affected women (Culley et al., 2013). Despite the fact that various theories have been proposed to explain the onset of endometriotic lesions, the etiopathology of endometriosis fails to explain. Sampson’s explanation of retrograde menstruation is the most widely recognized of the proposed theories. Sampson’s theory proposed that endometriosis is caused due to the retrograde flow of endometrial cells/debris through the fallopian tube into the peritoneal cavity during menstruation. Admittedly, approximately 80–90% of women undergo retrograde menstruation; however, only around 5–10% of women develop EMs (Cramer and Missmer, 2002; Sasson and Taylor, 2008). This distinction between the two proportions indicates that the refluxed endometrial tissue is not the only reason for the development of endometriosis. There may be additional responses to the ectopic endometrium in the local environment that enables their implantation and further development into ectopic lesions. Moreover, increasing evidence indicates that the successful establishment and survival of ectopic implants in the ectopic microenvironment (peritoneal cavity or pelvic organs) require tumour-like migration and invasion, adhesion formation, vascularization, fibrosis, and neuronal infiltration (Rocha et al., 2013; Filippi et al., 2016; Guo et al., 2016).

Inflammation and angiogenesis at the site of the ectopic implant have been considered to involve in the development of ectopic lesion (Gazvani and Templeton, 2002; Laschke and Menger, 2007). Previous studies reported the high concentration of proinflammatory cytokines in the peritoneal fluid of endometrial patients (Gazvani and Templeton, 2002). Recent *in vitro* studies have also shown that ectopic lesion possesses the ability to produce endometriosis-related cytokines and/or regulators of cytokine production to facilitate systemic inflammation, as well as stimulate angiogenesis (Ahn et al., 2015; Khalaj et al., 2017). However, whether the observed high concentration of cytokines and peritoneal fluid is the cause or the consequences of Ems has yet to be further described. More importantly, it is still unclear which factors are regulating the inflammation and angiogenesis at the site of ectopic implantation caused by retrograde endometrium.

As a natural cargo, exosomes carry their critical biological information feature, including nucleic acids, proteins, and lipids (Ge et al., 2012; Zhu et al., 2018), to maintain cellular communication in the microenvironment. Tumor-derived exosomes have been shown to contribute to the progression and metastasis of cancer by transferring genetic molecules from their parent cells to recipient cells, thereby regulating the biological function of recipient cells (Azmi et al., 2013; De Toro et al., 2015). Recent studies on the implications of exosome in the development of endometriosis, especially in the ectopic microenvironment, has made significant advances in our understanding of endometriosis. The best example is that, in endometriosis, exosomes carrying specific gene molecules, such as miRNA and/or lncRNAs involved in inflammation, fibrosis, and angiogenesis, are engaged in the development of EMs (Harp et al., 2016; Wu et al., 2018; Zhang et al., 2019). However, the implication of the exosomal proteins in the establishment of endometrial lesions, and the pathophysiology of EMS have not been well characterized.

In this study, we observed that the ectopic endometrial cells-derived exosomes induced the migration capability of healthy endometrial cells and also contributed to the generation of angiogenesis and inflammation. It is thus hypothesized that the ectopic lesions may release exosomal protein to target the upcoming eutopic endometrium of reflux, as well as the surrounding microenvironment, thereby establishing an ectopic microenvironment with a “migration-inflammation-angiogenesis” loop conducive to the progression of endometriosis. 

## Materials and Methods

### Patients and Samples

All the clinical specimens including, endometrial tissue and vaginal secretion (leukorrhea), were collected from the patients who enrolled in the Affiliated Hospital of Ningbo Medical School of Ningbo University (Ningbo, China) (The general condition of patients in Supplementary Table1 S1 **)**. The primary normal endometrium samples were collected from the reproductive-age women undergoing surgery for other benign gynecological conditions (*n* = 10). Primary ectopic endometrial stromal cells were obtained from women with endometriosis, ovarian endometriosis, particularly (*n* = 6). The vaginal secretion (leukorrhea) was collected from corresponding endometriosis patients. For the control group, vaginal secretion specimens were obtained from patients without endometriosis and any other hormone-related disorders, and women with the normal menstrual cycle were admitted for physical examination. Ovarian epithelial cells were isolated from the ovarian tissue obtained from patients who underwent bilateral appendices hysterectomy excluded from endometriosis. All the subjects selected were women free from hormonal therapy for 3 months and had regular menstrual cycles. Samples obtained from all patients and controls were matched for the secretory phase of the menstrual cycle. Written informed consent was obtained from all women before the sample collection, and all procedures were approved by the ethical committee of the Affiliated Hospital of Ningbo Medical School of Ningbo University (Ningbo, China).

### Cell Culture

Primary normal endometrial stromal cells (NSCs), ectopic endometrial stromal cells (ESCs) were isolated from endometrial tissue after tissue digestion. Digested tissue was filtered through 100 and 40 μM mesh to collect the stromal cells. Cells were then incubated in DME/F12 medium (Gibco, United States) supplemented with 10% fetal bovine serum (FBS) (Gibco, United States), 1% penicillin (100 U/mL) and streptomycin (100 μg/ml) at 37°C and 5% CO2 in a humidified incubator. Human umbilical vein endothelial cells (HUVECs) were purchased from the cell bank of the Chinese Academy of Sciences (China) and were cultured in DMEM complete medium. Ovarian epithelial cells were cultured in DME/F12 medium supplemented with 10% FBS and 1% penicillin and streptomycin. All of the primary cells were cultured less than 14 days *in vitro* environment, and only passage 1 (P1) cells were taken for subsequent experiments.

### Exosomes Isolation and Identification

Exosomes were isolated from NSCs and ESCs culture supernatant, as well as patients’ vaginal secretion. NSCs and ESCs were incubated primarily in culture media with 10% exosomes-free FBS, which was obtained by 12 h ultracentrifugation of FBS at 100,000 g at 4°C. After 48 h of incubation, cell culture supernatants were collected, and exosomes were isolated as previously described (Lässer et al., 2012). Subsequently, exosomal protein concentrations were determined using bicinchoninic acid (BCA) protein assay kit (Thermo Scientific, United States) according to the manufacturer’s instructions. Allexosomal protein samples were standardized to the quantity of 10 ug for *in-vitro* and 20 μg for the *in vivo* experiment. Then, exosomal classical marker CD9, CD63, and CD81 (Cell Signalling Technology, Beverly, MA, United States) expression was measured using western blot analysis. The aliquots were stored at—80°C for subsequent experiments. The extracted exosomes and pellets were sent to Hibio Technology Co., Ltd., (Hangzhou, China) for transmission electron microscope (TEM) observation and validation, and the size distribution measurement analysis.

### Fluorescent Labeling of Exosomes

This assay was performed to identify the internalization of the exosomes from ESCs into NSCs. Briefly, isolated exosomes were re-suspended in 500 μl of PBS in a 1.5 ml microcentrifuge tube (Eppendorf, EP), and PHK26-Green (Umibio Science and Technology Co., Ltd., Shanghai, China) with a final concentration of 5 μg/ml was added to label the exosomes and incubated at 37°C for 30 min without shaking. Labeled exosomes were centrifuged at 10,000× g for 10 min, and the supernatant was carefully filtered with a 0.22-μM filter. PHK26-labeled exosomes were then co-cultured with NSCs and HUVECs for 24 h in a 6-well plate. After 24 h, NSCs were labelled with Coralite594-conjugated vimentin monoclonal antibody (CL594-60330, proteintech, China) for 1 h, room-temperature, in the dark, and the nuclei of HUVECs were labeled with DAPI. The cells were then prepared for immunofluorescence analysis, and the internalization of exosomes was subsequently observed under a laser scanning confocal microscope (Leica, TCS SP80).

### Migration and Wound-Healing Assay

Cells were pre-treated with exosomes (5 μg/ml) or with exosomes-free culture medium for 24 h. For the migration assay, approximately 4 × 10^4^ cells were seeded into the upper transwell chambers and incubated with an FBS-free culture medium, while the lower chambers were maintained in a culture medium with 10% FBS. After 24 h incubation, all cells transferred to the lower chambers were fixed with 2% methanol and stained with 0.5% crystal Violet. Positive staining cells from six representative fields of chambers in each group were photographed and counted under a microscope (CKX40, Olympus, Japan). For wound healing assays, cells were pre-treated with exosomes and exosomes-free culture medium as previously described, equal amounts of cells were plated into six-well plates. Cells monolayer were scratched with a pipette tip to draw a gap between cells on the bottom of the plates, cell migration was observed, and images were taken at 0 h, 24 h, and 48 h time points. Subsequently, the number of migrating cells was quantified using Image-pro plus (PPI, United States).

### Matrigel Tube Formation Assay

Human umbilical vein endothelial cells (HUVECs) were pre-treated with both NSCs and ESCs derived exosomes (5 μg/ml) or with PBS as a control for 24 h. About 2.5×10^4^ HUVEC cells were seeded in growth factor-reduced Geltrex Basement Membrane Matrix (BD, United States) (40 μl per well, cured at 37°C for 2 h before the test) on a 96-well plate and incubated up to 2 h at 37°C with 5% CO^2^. The formation of the tube was observed under the microscope, and images were captured. Formative total master segments length and Branch points were determined using Angiogenesis Analyse app of ImageJ software. Results are presented as the mean and standard errors.

### Western Blotting

The total proteins of cells and exosome pellets were lysed with RIPA buffer supplemented with proteinase inhibitors (Beyotime Biotechnology, China), as per manufacturer’s protocols, and centrifuged at 14,000× g for 10 min at 4°C. Protein concentrations were determined using the BCA protein assay kit, as previously described. Protein samples were (25 μg if extracted from cells and 35 μg if extracted from exosomes) separated by 8–12% sodium dodecyl sulfate-polyacrylamide gel electrophoresis (SDS-PAGE), and transferred onto 0.22 μM polyvinylidene difluoride (PVDF) membranes. Membranes were blocked in 5% skim milk TBS-T solution for 1 h at room temperature and incubated with primary antibody overnight at 4°C. The following primary antibodies were used in this blotting: anti-CD9 (1:1,000; CST, United States), anti-CD63 (1:1,000; CST, United States), anti-CD81 (1:1,000; CST, United States), anti-GAPDH (1: 3,000; Bios, China), anti-VEGFR (1:1,000; CST, United States), anti-pVEGFR2 (1:1,000; CST, United States), anti-PDGFR (1:1,000; CST, United States), anti-pPDGFR (1:1,000; CST, United States), anti-ERα (1:1,000, Abcam, United States), anti-Gα_13_ (1:200, Santa-Cruz, United States), anti-RhoA (1:200, Santa-Cruz, United States), anti-ROCK-2 (1:200, Santa-Cruz, United States), anti-MSN(1:500, proteintech, China), anti-MMP9(10375-2-AP, 1:1,000, proteintech, China), Epithelial-Mesenchymal Transition (EMT) Antibody Sampler Kit (#9782, CST, United States), followed by incubation with appropriate horseradish peroxidase-conjugated secondary antibodies at room temperature for 2 h. Subsequently, immune-reactive protein bands were visualized with chemiluminescence reagents (CST, United States), followed by imaging on an electrophoresis gel imaging analysis system (GENE, United States).

### RNA Extraction and Quantitative Real-Time Polymerase Chain Reaction (qRT-PCR)

Total RNA was extracted from cultured cells and purified exosomes using TRIzolTM reagent (Life Technologies, United States), following the manufacturer’s instructions. The extracted RNA was re-suspended in 20–30 μl RNase-free DEPC water and stored at—80°C. 500 ng of total RNA was used for qRT-PCR analysis. The cDNA was synthesized using a transcription kit asper the manufacturer’s instructions (CWBio, Beijing, China). The qRT-PCR analysis was conducted using the SYBR Green PCR Kit (CWBio, Beijing, China) on an Applied Biosystems 7,500 Real-Time PCR System and associated software (Applied Biosystems, United States). After the initial denaturation step at 95°C for 10 min, three-step amplification was performed (95°C for 15 s, 60°C for 35 s) for 40 cycles. Relative expression levels of mRNAs were calculated with 2–ΔCt formulaor 2–ΔΔCtusing *GAPDH* (glyceraldehyde-3-phosphate dehydrogenase) as an internal control. Specific mRNA primers used for qRT-PCR are presented in (Supplementary Table S2).

### LC-MS/MS Analysis and Bioinformatics Analysis

TheWhole EVs Lysate from NSCs and ESCs were run for 20 min on a 4–20% SDS-PAGE system to separate proteins from lower molecular weight contaminants. The entire protein region of the gel was excised and subjected to in-gel trypsin digestion after reduction with dithiothreitol and alkylation with iodoacetamide. Peptides eluted from the gel were lyophilized and re-suspended in 25–50 μl of 5% acetonitrile(0.1% Formica acid) at 4 μl/min for 4 min onto a 100 μM I.D. Fused-silica pre-column packed with 2 cm of 5 μM nano viper (Thermo, United States). Peptides were eluted at 300 μl/min from a 75 μM I.D. gravity-pulled analytical column, packed with 25 cm of 3 μM Magic C18AQ particles using a linear gradient from 5 to 35% of mobile phase B (acetonitrile + 0.1% formic acid) in mobile phaseA (water + 0.1% formic acid) over 60 min. Ions were introduced by positive electrospray ionization *via* liquid junction at 1.4–1.6 kV into a Q Exactive hybrid mass spectrometer (Thermo Scientific). Mass spectra were acquired over m/z 300–1750 at 70,000 resolution (m/z 200) with an AGC target of 1 × 10^6^, and data-dependent acquisition selected the top 10 most abundant precursor ions for tandem mass spectrometry byHCD fragmentation using an isolation width of 1.6 Da, max fill time of 110 ms and AGC target of 1e^5^. Peptides were fragmented by normalized collisional energy of 27, and fragment spectra were acquired at a resolution of 17,500 (m/z 200).

A total of proteins in NSCs and ESCs exosomes were input to FunRich software (3.1.3) for GO (Gene Ontology) enrichment analysis (Gotz et al., 2008). The gene symbols retrieved from the UniProtKB accession number were mapped to cellular components, molecular function, and biological processes items. The protein comparison between NSCS exosomes and ESCs exosomes using the iBAQ method, the fold change of the protein was calculated by the logarithm of the ratio of individual protein values normalized to the average of the corresponding iBAQ values in the NSCs exosomes (*n* = 2) and ESCs exosomes (*n* = 2). The significant expression threshold was defined as a criterion of significance value < 0.05 and fold change > 2 (log2 FC > 1 or < -1). Cytoscape software and the KEGG pathway were used to analyze, integrate, and categorized the data.

### ELISA Detected the Expression of Moesin in Small Extracellular Vesicles Derived From Vaginal Secretion

According to the manufacturer’s instructions, the small extracellular vesicles collected from vaginal secretion were assayed by moesin ELISA (Cloud-Clone Corp., China). Exosomes’ protein concentrations were uniformly in 10 ug to be detected.

### Zymography for Detecting the Activity of MMP9

The exosomal proteins were lysated and then separated by 10% acrylamide separation gel (containing 1 mg/ml of gelatin). Because of divalent metal ions in the buffer system, MMP9 was hydrolyzed in the gelatin. After electrophoresis, the gel was stained with Komas Brilliant Blue for 30 min at room temperature and then was decolored overnight by decolorization solution.

### Transfection of ESR1 and MSN siRNA *in vitro*

ESCs were seeded in the 10 cm dish at 80% confluency and incubated in a culture medium supplemented with exosomes-free FBS for 24 h. ESCs were then transiently transfected with moesin siRNA (MSNi) 20 nm (Genepharma Cop., Shanghai, China) with lipofectamine‐2000TM (Invitrogen, United States). After 24 h of transfection, the cell culture supernatants were collected for three consecutive days. Exosomes were isolated from ESCs supernatants, and western blot analysis was performed to confirm the expression level of moesin in exosomes. Then, NSCs were incubated with exosomes^MSNi^ and non-transfected ESCs for 24 h and were prepared for subsequent experiments.

ESCs were seeded in the 6-wells plate at 60% confluency and transfected with ESR1 (estrogen receptor 1) siRNA 20 nm (Invitrogen, United States) with lipofectamine iMax (Invitrogen, United States). After 48 h of transfection, added estrogen (Meilun, Dalian, China) 10, 50, 100 nm into cell culture and collected cell protein after 24 h treatment.

### Mouse Model of Endometriosis

All animal experiments were approved by the committee on the Ethical Use of Animals of Medical School of Ningbo University. Experimental mice were kept following the Guide for the Care and Use of Laboratory animals, ina well-controlled, pathogen-free environment with regulated cycles of light/dark (12 h/12 h, 23–25°C), and given 2 weeks of adaptation before any experiments were conducted.

Briefly,35femalenude mice (Balb/c, 5–6 weeks old), were divided randomly into seven subgroups (PBS control, normal endometrial cells, ectopic endometrial cells, ectopic endometrial cells + MSN^ncRNA^, ectopic endometrial cells + MSN^siRNA^, normal endometrial cells + ESCs-derived exosomes, normal endometrial cells + ESC-derived exosomal MSN^siRNA^) before implantation, with five mice in each group. All siRNA-treated cells were interfered with for 48 h before injection into mice. Exosomes secreted by ectopic endosomal cells were collected. We knocked down MSN mRNA expression in exosomes by electro-transferring siRNA and co-incubated with normal endosomal cells for 48 h. All interfered cells were quantified to determine the effect of interference by RT-qPCR. Prepared cell suspensions for six different groups (about 1 × 10^7^/ml cells in each suspension) were administrated by peritoneal injection into each mice on day 0. Mice were weighed and recorded every 2 days. After 20 days of observation, mice were then sacrificed and dissected to determine the heterotopic inoculation of endometrial tissue on the intestinal wall. We fixed the ectopic lesion in formalin for subsequent immunohistochemistry and immunofluorescence, while we isolated small intestinal tissue 5 cm around the ectopic lesion to detect the expression of inflammatory factors.

### Immunohistochemistry

Formalin-fixed paraffin-embedded (FFPF) mouse ectopic endometrial tissues were sectioned into 4 μM thick tissue slices. Slices were deparaffinized in xylene, rehydrated through graded ethanol, and boiled for 10 min in citrate buffer (10 mm, pH 6.0) for antigen retrieval. Endogenous peroxidase activity was suppressed by exposure to 3% hydrogen peroxide for 10 min. Tissue slides were then blocked with 5% BSA (bovine serum albumin; Boster Bioengineering, Wuhan, China), and incubated overnight at 4°C with the following primary antibodies:anti-MMP9 (Absin, China) and anti-VEGFR2 (CST, United States), followed by incubation with corresponding secondary antibody for 20 min. Slides were visualized, adding DAB (3,3′-diaminobenzidine) substrate, counterstained with hematoxylin, and mounted for observation under the microscope.

### Immunofluorescence

Deparaffinization and antigen retrieval was performed as mentioned above. Tissue sections were then permeabilized by 0.1% TritonX-100 (Sigma Aldrich) for 10 min. Unspecific bindings were blocked by using PBS + 8% Normal goat serum for 1 h at RT. Tissue sections were incubated overnight at 4°C with the following anti-pVEGFR2 (1:200, CST, United States). On the second day, slides were washed 3 times in PBS and incubated with the secondary antibody: Goat anti-rabbit antibody 647 (1:500, Abcam, United States) for 1 h at RT in dark. Finally, sections were mounted using Fluorescent Mounting Medium and visualized under the microscope (Leica, Germany).

### Statistical Analysis

The statistical analysis was performed using GraphPad Prism version 6.0 (GraphPad) and SPSS software (version 20.0; IBM Corp., Armonk, NY, United States). Comparisons between two group experiments were performed using a two-tailed Student’s t-test, while multiple comparisons between the groups were analyzed using a one-way analysis of variance (ANOVA), followed by the Student Newman-Keuls test. All experiments were performed in triplicate, and the quantification of results was presented as the mean ± standard deviation. *p* < 0.05 was considered statistically significant.

## Results

### Identification of Exosomes Derived From Endometrial Cells and Vaginal Discharge

**FIGURE 1:**
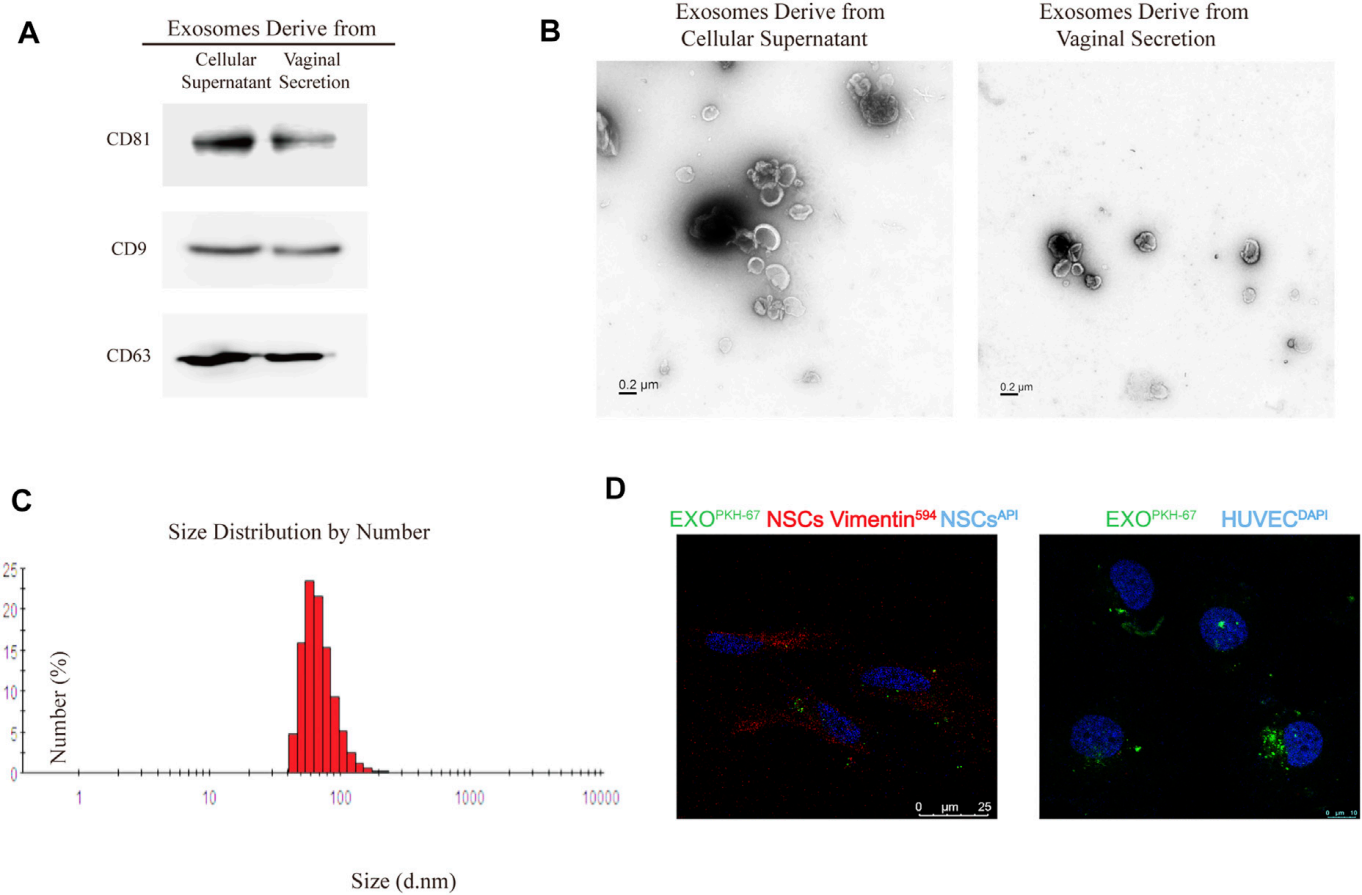
Identification of exosome characteristics. **(A)** Western blot analysis of exosomal marker CD63, CD81, and CD9 in exosomes from primary cellular supernatant and vaginal secretion. CD9 did not express in exosomes derived from vaginal secretion. **(B)** Transmission electron microscope (TEM) of exosomes. **(C)** The average size of exosomes is 95.5 nm. **(D)**. PKH67 staining of exosomes internalization by Normal endometrial stromal cells (NSCs) and HUVECs were observed by the confocal microscope, anti-vimentin was the NSCs marker, HUVECs nuclei labeled with DAPI.

Exosomes were isolated from ESCs incubated for 48 h in exosomes-free culture medium and patients vaginal discharge by ultracentrifugation as previously described (Yu et al., 2019). CD9, CD63, and CD81 as recognized exosome-specific markers were used to confirm isolated exosomes using western blot analysis (Figure 1A). The TEM images showed that the majority of exosome vesicles were round-shaped and membrane-bounded (Figure 1B). Isolated exosomes were further characterized by a particle size analyzer, which indicated exosomes as rounded particles with a diameter range 60–150 nm (the average diameter of 95.5 nm) in size (Figure 1C). Furthermore, we tested whether these exosomes could be internalized by NSCs and HUVECs to effect their function in the uterine or peritoneal ectopic microenvironment. Briefly, exosomes were labeled with PKH67, a lipophilic fluorescent dye. A laser scanning confocal microscope assessed the internalization of PKH67-labeled exosomes within NSCs and HUVECs. We observed that PHK67-labelled exosomes were internalized into the cytoplasm of NSCs and HUVECs (Figure 1D). This result confirmed that exosomes derived from ESCs could be taken up and internalized by NSCs and HUVECs.

### ESCs-Derived Exosomes Induced Normal Endometrial Stromal Cells Migration, Angiogenesis, and Upregulated Inflammatory Cytokines Expression in Ovarian Cells

**FIGURE 2:**
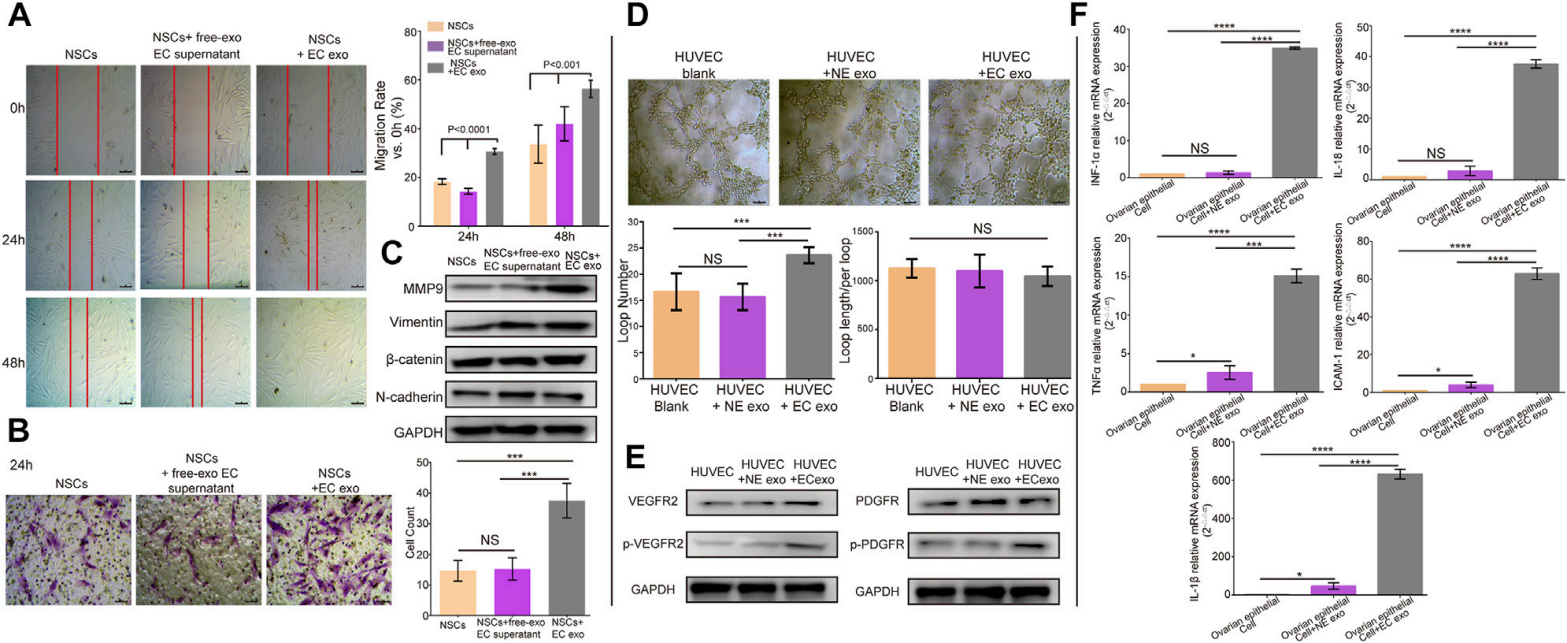
ESCs-derived exosomes induced migration, angiogenesis, and upregulated inflammatory. **(A)** Wound-healing assay to verify the effects of exosomes on normal endometrial stromal cells (NSCs) migration. **(B)** Transwell assay to evaluate the migration ability, along with the quantification of migrated cells and migration rate. **(C)** Western blotting showed ectopic exosomes overexpressed MMP9, but there were no changes in the expression level of EMT- related proteins. **(D)** A tube formation assay along with quantification of the number of loops formed and loop length/per loop. **(E)** Western blotting analysis assessed the expression of VEGFR2, p-VEGFR2, PDGFR and p-PDGFR. **(F)** RT-qPCR tested the expression of inflammatory cytokines, data were expressed as mean ± SEM, NS, no significance, ****p < 0.0001.

To identify our hypothesis that ESCs-derived exosomes can affect the NSCs and the cells surrounding the ectopic lesion. We added exosomes isolated from ESCs into NSCs and subjected them to wound-healing and migration assay to evaluate cell motility and migration. Cells treated with exosomes-free standard culture medium were used as control. The wound-healing assay showed that ESCs- derived exosomes significantly improved the mobility of NSCs and exosomes-free standard culture medium group, the migration rate of NSCs was 18.33% compared to NSCs co ectopic exosomes, which were 30.67% in the first 24 h (t = 3.48, *p* = 0.0083. After 48 h, the migration rate of NSCs increased to 33.67%, while the rate of NSCs co ectopic exosomes was increased to 56.33% (t = 6.39, *p* = 0.0002), there was no statistical difference in migration results between the exosome-free EC medium group and the NSCs group, with lower migration capacity than the NSCs co EC exo group (*p* < 0.001) (Figure 2A). Transwell assay further confirmed that the ESCs-derived exosomes promoted NSCs migration, in which, the number of migrated NSCs was more than two times higher in the ESCs-derived exosomes group (37.59 ± 1.372) compared to the control group and the exosome-free EC medium group (14.61 ± 0.7968,15.01 ± 0.1248) (t = 14.68, *p* < 0.0001; t = 13.43,*p* < 0.0001) (Figure 2B). We also examined the no effect of ECSs-derived exosomes on apoptosis of NSCs cells by cell flow apoptosis assay (Supplementary Figure S1). We assessed the Epithelial-Mesenchymal Transition (EMT) related proteins, including Vimentin, N-cadherin, E-cadherin, β-catenin, Slug, Snail, ZEB1, ZO-1, and Claudin-1 by western blotting. Interestingly, the result showed that only Vimentin, β-catenin, and N-cadherin were expressed in the primary endometrial cell. Moreover, the expression of these three proteins showed no change after treated with ectopic exosome. However, the expression of MMP9, which is one of the classic MMP family proteins, was increased treated with NSCs co ectopic exo group (Figure 2C). The statistical results for WB were in Supplementary Figure S3.

Furthermore, we conducted the tube formation assay to evaluate the effect of ESCs-derived exosomes on angiogenesis. HUVECs were incubated with exosomes isolated from ESCs and NSCs. We observed that HUVECs treated with ESCs-derived exosomes showed a significant increase in the number of generated loops formed compared to the control group and NSCs exo group (t = 3.116, *p =* 0.034, *p* = 0.045), and no significant difference in the loop length/per loop in three groups (Figure 2D). We further examined the expression of angiogenesis-related proteins in HUVECs treated with ESCs derived exosomes by western blotting. Angiogenesis is regulated by multiple signaling pathways, of which VEGF and its ligand VEGFR are the major regulatory proteins. However, when the VEGF and VEGFR pathways are inhibited under certain circumstances, other angiogenesis-related proteins such as platelet-derived growth factor (PDGF)/platelet-derived growth factor receptor (PDGFR) and fibroblast growth factor (FGF) and its receptor (FGFR) will be activated and consequently trigger angiogenesis. In this study, we, therefore, chose VEGFR2 and PDGFR as indicators of angiogenesis (Zhao and Adjei, 2015). We found that there was no significant difference in the expression level of PDGFR, whereas the VEGFR2 and p-VEGFR2 expression level was significantly higher in HUVECs treated with ESCs-derived exosomes than in the control group and NSCs group. The level of P-PDGFR expression was also increased, which was less than P-VEGFR2 (Figure 2E). This provided further evidence to support the effect of ESCs-derived exosomes in regulating angiogenesis. The statistical results for WB were in Supplementary Figure S3.

Moreover, endometriosis has known to be an inflammatory disease. Previous work showed that the expression level of proinflammatory cytokines was increased in peritoneal fluid (Yu et al., 2021) (Arosh et al., 2022). Considering that ESCs-derived exosomes may contribute to the onset of inflammation in the ectopic environment, we assessed the gene expression level of proinflammatory cytokines in normal ovarian epithelial cells by real-time qPCR analysis. Normal ovarian epithelial cells were treated with and without ESCs-derived exosomes and NSCs-derived exosomes for 24 h. We observed that expression levels of proinflammatory cytokines, including IL-1β, IL-18, INF-1α, TNF-α, and ICAM-1, were significantly higher in normal ovarian epithelial cells treated with ESCs-derived exosomes compared to the control group and the NSCs-derived exosomes group (*p* < 0.0001) (Figure 2F). Taken together, these results suggested that exosomes contributed to the development of endometriosis.

### Characterization of Exosomal Protein Derived From Ectopic Endometrial Stromal Cells Versus Normal Endometrial Stromal Cells by Mass Spectrometry

**FIGURE 3:**
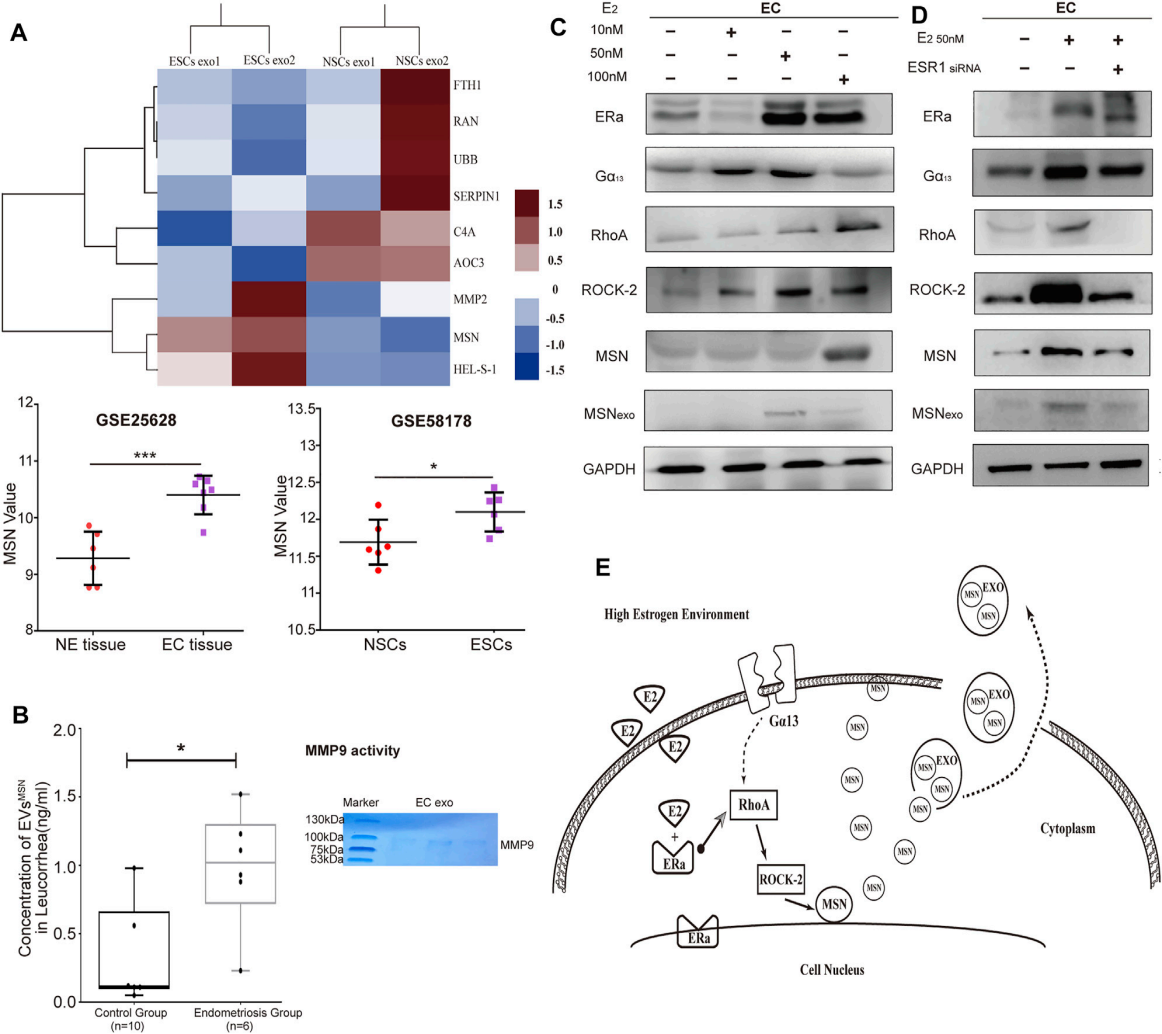
The proteome profile of NSCs- and ESCs exosomes. **(A)** Heatmap of six significantly up-regulated proteins and three down-regulated proteins in NSCs exosomes (*n* = 2) versus ECSs exosomes (*n* = 2), with depletion depicted in blue and enrichment in red. GEO dataset for RNA high-throughput sequencing results of endometriosis (GSE25628 for tissue and GSE58178 for primary stromal cells), which showed that MSN value in ectopic endometrial tissues (primary stromal cells) were higher than those in the normal group (GSE25628, ***p < 0.001; GSE58178, *p < 0.05). **(B)** Elisa assay to detect the expression of MSN in vaginal secretion. Data were expressed as mean ± SEM, *p < 0.05. Acrylamide-gelatin electrophoresis detected the activity of MMP9 in EVs. **(C)** Three concentration of estrogenwas treated with NSCs. The expression of RhoA, ROCK-2, MSN, and Gα_13_ was tested by western blot. **(D)** Western blot analysis to assess ERα recruited RhoA/Rock-2/MSN signaling pathway, but Gα_13_ displayed no significant change. **(E)** The pattern diagram of Era/RhoA/Rock-2/MSN signaling.

To characterize the protein profilesof NSCs and ESCs exosomes, we performed MS analysis in exosomes isolated from ESCs and NSCs supernatants. Proteomic analysis revealed a total of 155 proteins in exosomes from both groups, with 139 proteins were differently expressed in ESCs exosomes (Supplementary Table S1). Gene Ontology (GO) classification using Funrich function enrichment analysis (Lässer et al., 2012) identified the biological processes that enriched for molecular functions (MFs). This includes the regulation of the biological process, biological regulation, cellular process, metabolic process, and response to the stimulus. To examine the differentially expressed proteins between NSCs and ESCs exosomes, we calculated two factors that constitute the fold change of protein expression and LFQ intensity between two groups, and a shuttle plot was generated. A total of three proteins were significantly up-regulated in ESCs exosomes with criteria of *p* < 0.05, while six proteins were remarkably down-regulated in ESCs exosomes (*p* < 0.05) (Supplementary Table S3). A heatmap was generated to highlight the differently expressed proteins in NSCs and ESCs exosomes. As there were only two samples per group in the exosomal protein spectrum assay, to avoid the problem of accuracy of the results, we searched the GEO dataset for RNA high-throughput sequencing results of endometriosis (GSE25628 for tissue and GSE58178 for primary stromal cells), which showed that the mRNA expression levels of MSN in ectopic endometrial tissues (primary stromal cells) were higher than those in the normal group (GSE25628, *p* < 0.001; GSE58178, *p* < 0.05) (Figure 3A). According to the KEGG pathway, we found a proteoglycan-related molecule-- Moesin (MSN), which is one of the up-regulated proteins in ESCs exosomes. MSN is thought to be involved in angiogenesis, migration, and cell adhesion. Therefore, we assessed the expression of moesin in exosomes from the vaginal secretion from endometriosis patients (*n* = 6) and non-endometriosis patients (*n* = 10). The result indicated that the average protein expression of MSN was 0.3217 ng/ml in the control groups, which was significantly lower than endometriosis group (average was 0.9833) (*p* = 0.0179). We also detected the activity of MMP9 in ectopic stromal cell-derived exosomes by enzymatic spectroscopy, where we could observe bands at the location of MMP9 in acrylamide-gelatin electrophoresis, indicating that MMP9 in EVs is biologically active. (Figure 3B).

A previous study reported that Estrogen (E2) regulates MSN expression by recruiting Gα_13_—dependent pathway to RhoA and ROCK-2 (Sanchez et al., 2009). Since endometriosis is associated with high Estrogen concentration (Othman et al., 2021), we examinedthe effect of E2 on MSN expression. Treatment with E2 (10, 50, 100 nm) induced the expression of estrogen receptor *α* (ERα), led to the activation of the Gα_13_-RhoA-ROCK-2-MSN signaling pathway, which resulted in the upregulation of MSN expression in endometriosis (Figure 3C). To examine how ERαrecruits the signaling of E2 to Gα_13_dependent pathway to regulate MSN, we silenced ERα with small interfering RNA (siRNAs). ERα expression was decreased after transfection with ERα siRNA. Western blot analysis showed that ERα silencing did not affect ERα expression, whereas RhoA, ROCK-2, MSN, and exosomal MSN expression were significantly decreased (Figure 3D). This suggested that ERαplays an important role in recruiting this signaling pathway to MSN, although the impact of ERα on Gα_13_expression needs further exploration. In addition, we also identified the effects of other estrogen receptors, including ERβ and GPR30, in mediating the signaling of E2 to MSN (Data not shown). We observed that ERα had a significant effect on recruiting signaling of E2 to MSN (Figure 3E).

### Exosomal Moesin Upregulated the Expression of MMP9, VEGFR2/p-VEGFR2 and Inflammatory Factors

**FIGURE 4:**
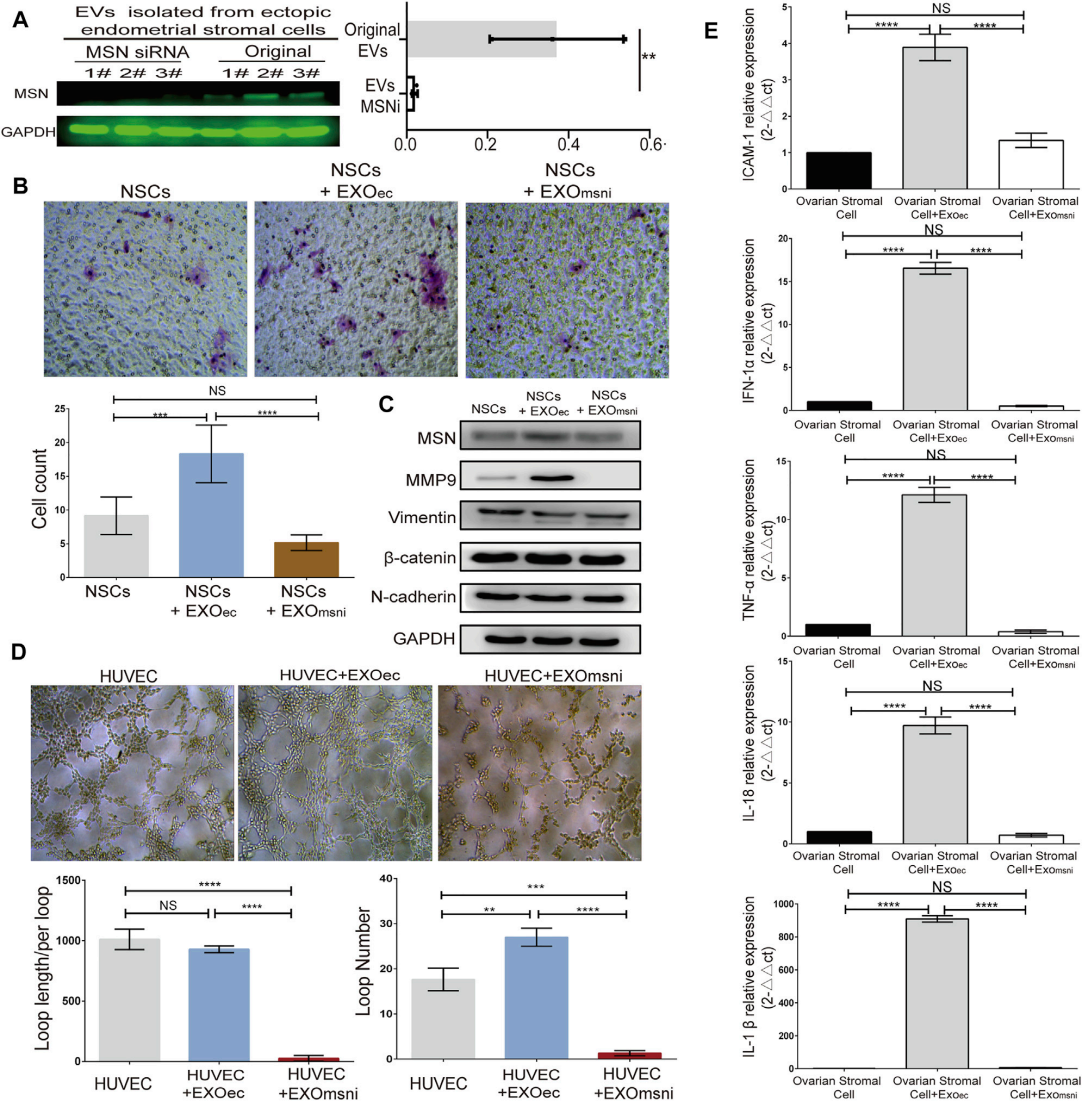
Exosomal Moesin induced the expression of MMP9, VEGFR2 and inflammatory factors. **(A)** Western blotting detection of MSN in exosomes from ESCs after siRNA transfection. **(B)** Transwell assay and quantification of the migration of NSCs treated with exosome + MSNi. **(C)** Western blotting detection of the expression of MSN, MMP9, and EMT-related proteins. **(D)** Tube formation after exo + MSNi and ectopic exosomes treatment. **(E)** RT-qPCR analysis of inflammatory cytokines, data were expressed as mean ± SEM, NS, no significance, **** *p* < 0.0001. EXO, ectopic stromal cells derived exsomes. MSNi, MSN siRNA。.

Through MSNsiRNA transfection, we down-regulated the expression level of MSN in three primary ectopic stromal cells, and western blot analysis showed that the exosomal MSN was significantly decreased from those three transfected ECSs (Figure 4A). To assess how exosomal MSN regulated NSCs migration, angiogenesis, and inflammation of the surrounding tissues (ovarian epithelial cells). ESCs exosomes and exosome^MSNi^ were co-cultured with NSCs, HUVEC, and ovarian epithelial cells, respectively. Transwell assay showed that the number of NSCs was notably decreased after the treatment with exosome^MSNi^ (mean ± SD: 5.167 ± 1.169) compared to NSCs + ectopic exosome group (mean ± SD: 18.33 ± 4.274) (t = 7.546, *p* < 0.0001), whereas no statistical difference was observed between NSCs group and NSCs + ectopic exosome^MSNi^ group (Figure 4B). In the meantime, the expression of MMP9 and EMT related proteins were detected by Western blot, the result revealed that the expression of MMP9 and MSN were significantly reduced in NESC + ectopic exosome^MSNi^ group, while all the detectable EMT related proteins expression remained unchanged in these three groups (Figure 4C). The statistical results for WB were in Supplementary Figure S3. In the tube formation assay, lumen formation was inhibited in the NESC + ectopic exosome^MSNi^ group (Figure 4D). Finally, we found that mRNA expression of *IL-18*, *IL-1*, *TNF-α*, *INF-α1*, and *ICAM1* were prominently decreased in ovarian epithelial cells treated with exosomeMSNi (*p* < 0.0001) (Figure 4E).

### Exosome^MSNi^ Inhibited the Onset of Intestinal Heterotopic Mass and Peripheral Inflammation *in vivo*.

**FIGURE 5:**
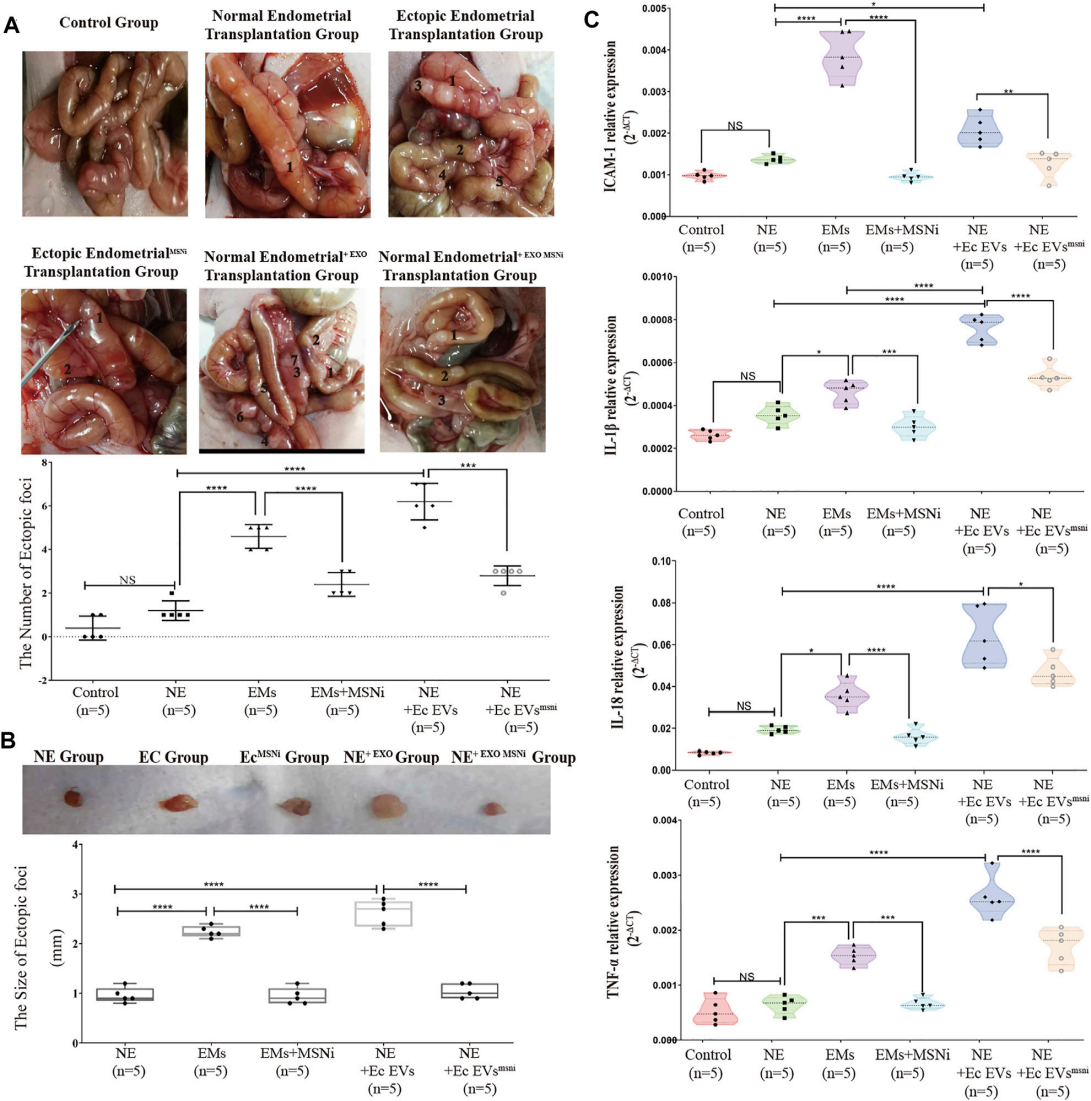
**(A)** The model of heterotopia mouse was established by intraperitoneal injection. Ectopic stromal cells and normal endometrial stromal cells + ectopic exosomes notably increased the number of ectopic foci compared with the normal endometrial stromal cells group and control group (*p* < 0.0001), while MSNi and exosomal MSNi inhibited this phenomenon (*p* < 0.0005). **(B)** The size of ectopic implant tissue. Ectopic exosomes could increase the size of ectopic tissue the same as the ectopic cells, but exosomal MSNi and ectopic cells + MSNi decreased the size (*p* < 0.0001). **(C)** The expression of inflammatory cytokines: IL-1β, ICAM-1, IL-18, and TNF-αin the small intestinal tissue surrounding the heterotopic foci. Data were expressed as mean ± SEM, NS, no significance, **** *p* < 0.0001.

To verify the effect of exosome^MSN^ on endometrial tissue *in vivo*, we divided the primary cells into seven groups: PBS (as the control group), normal endometrial cells, ectopic endometrial cells, ectopic endometrial cells treated with MSNnc, ectopic endometrial cells treated with MSNi, normal endometrial cells treated with exosomes derived from ectopic endometrial cells, and endometrial cells treated with exosomeMSNi. We transplanted these cells into the nude mice by intraperitoneal injection. Mice were weighed every 2 days and found no difference between the groups (data not shown). After 20 days, we culled the mice, and observed that mice in all experiment groups had heterotopic tumor growth inside the small intestine except the PBS control group. There were significant differences in the number and size of the masses formed in each group. The number and size of masses formed were increased in endometrial cells treated with ectopic exosomes group, while there was a reduction in the number and size of masses formed in the exosomal MSNi group (*p* < 0.0001) (Figures 5A,B). The number and size of masses in the ectopic groups were also notably higher than that in the normal endometrial group (*p* < 0.0001) (Figures 5A,B), which was the same as seen in the endometrial cells treated with ectopic exosomes groups. Similarly, the intestinal mass in the ectopic group was remarkably improved after MSN down-expression (*p* < 0.0001) (Figures 5A,B). We simultaneously assayed the effect of the ectopic endometrial cells treated with MSN NCsiRNA group on ectopic lesion formation which showed that MSN NCsiRNA did not affect the size and weight of the ectopic lesions without statistical difference from the ectopic endometrial cells group. In contrast, the lesion size and weight were larger than that of the normal endometrial cells group (Supplementary Figure S2). To assess the extent of peripheral inflammation, we selected the small intestine tissues with 5 cm of the tumor and analyzed the expression of inflammatory factors. We observed that both ectopic endometrial cells and the normal endometrial cells treated with ectopic exosomes increased the expression of IL-18, IL-1beta, TNF-a, and adhesion factor ICAM-1, however, the knockdown of MSN reversed this change(Figure 5C).

### Exosome^MSNi^ Decreased the Expression of MMP9 in Foci and the Formation of the Vascular Lumen *in vivo*

**FIGURE 6:**
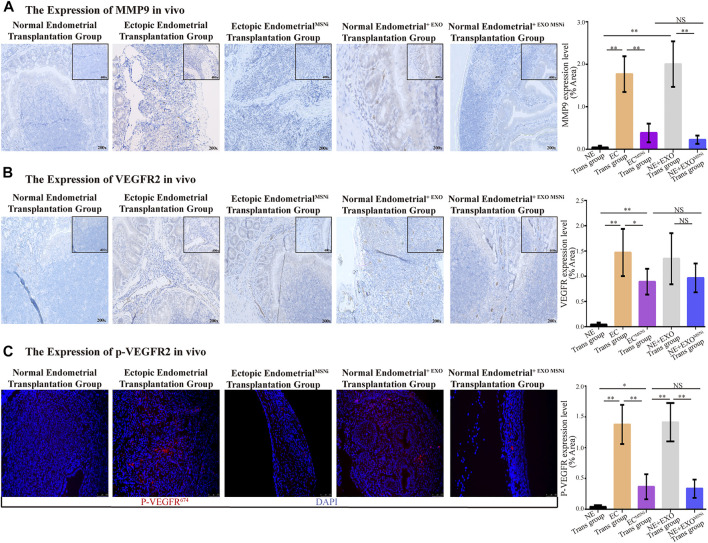
Exosome^MSN^ increased the expression of MMP9, VEGFR2 and p-VEGFR2 *in vivo*. **(A)** MMP9 expressed in ectopic implant tissues detected by IHC. **(B)** VEGFR2 expressed in ectopic implant tissues detected by IHC. **(C)** Immunofluorescence of p-VEGFR2 in ectopic foci. The result was consistent with VEGFR2. MSNi: MSN siRNA transfection; Microscopic magnification: ×200. **, *p* < 0.001; *, *p* < 0.05; NS, no significance.

To probe into the expression of MMP9, VEGFR2 and p-VEGFR2 in ectopic foci. Immunohistochemistry (IHC) staining showed that MMP9 presented more significant expression on the edge of the ectopic mass in the ectopic endometrial cells group, and the normal endometrial cells treated with exo^MSN^ group. In contrast, in MSN knockdown groups, the expression of MMP9 was notably decreased, whereas there was no difference in normal endometrium (Figure 6A). Meanwhile, large vascular lumens were formed in the tissue, and VEGFR2 was expressed mainly in lumens in both the ectopic and endometrial cells treated with ectopic exosomes. Although VEGFR2 was also expressed in the ectopic endometrial^MSNi^ group and normal endometrial cells treated with exosome^MSNi^, most of the lumens where VEGFR2 was expressed were small and partially closed (Figure 6B). We detected the expression of p-VEGFR2 in heterotopic tissue by IF staining. Similarly, we found that p-VEGFR2 was predominantly expressed in the vascular cavity in the ectopic endometrial cells group and the normal endometrial cells treated with exo^MSN^ group (Figure 6C).

## Discussion

Endometrium with abnormal migration ability is the fundamental contributor to the formation of an endometriotic lesion in the ectopic area. Endometriosis is associated with the reflux of endometrial tissue into the peritoneal cavity by traveling through the fallopian tubes during menstruation. It attaches, forms an implant, and proliferates at the ectopic site (Simpson et al., 2003). However, whether the microenvironment of the ectopic implantation is conducive to adhesion and colonization of the upcoming endometrial cells remains unknown. Sun et al. (2019) reported that eutopic endometrial cells-derived exosomes promote neuro-angiogenesis in endometriosis (Sun et al., 2019). In this study, we studied how the ectopic endometrial cells utilized exosomes to build up a “migration-inflammation-vascularisation” loop to facilitate the development of endometriosis.

Exosomes are small membrane vesicles that range in size, between 30 and 120 nm, and are released by the vast majority of cell types. Exosomes are detectable in different types of body fluids, including blood, saliva, urine, and uterine fluid (Vlassov et al., 2012; Burns et al., 2014). In Ouattara LA study on exosomes in vaginitis, exosomes were isolated from patients’ vaginal secretion for follow-up detection (Ouattara et al., 2018). In this study, we isolated exosomes from ESCs and NSCs, and subjected them to Mass Spectrometry analysis (3cases, lost 1case). We found that six proteins were highly expressed in ESCs exosomes, and three proteins were expressed at a relatively lower level. After further analysis, we found that Moesin (MSN) showed a strong correlation with migration, angiogenesis, and inflammation. Besides, MSN was reported to crosstalk the heparan sulfate proteoglycans (HSPGs) pathway with MMP9 through KEGG analysis. Interestingly, MMP9 is associated with cell migration, vascular formation, and inflammation as well (Hernandez et al., 2018; Li et al., 2019; Liu et al., 2019).

MSN is a member of the ERM(ezrin, radixin, and moesin) protein family discovered in 1992, and other members of ERM protein include ezrin, radixin, and merlin (Ivetic and Ridley, 2004). ERM proteins are bridge molecules that cross-link the cortical actin cytoskeleton with the integrin on the plasma membrane. By mediating the connection between the cytoplasmic membrane and actin cytoskeleton, ERM proteins play a critical role in cell growth, movement, migration, mitosis, and signal transduction (Polesello and Payre, 2004). In gynaecological oncology, high expression of ERM is known to promote the proliferation and migration of tumour cells, and even facilitate the formation of the blood vessel. Z Chen et al. reported that activation of ezrin was increased in ovarian epithelial carcinoma (OVCA), and related to OVCA metastatic process (Chen et al., 2001). Paulette M. et al. showed that high expression of MSN in endometrial adenocarcinoma is associated with grade and subtype (Mhawech-Fauceglia et al., 2012). In this study, we identified the role of exosomes derived from ESCs, specifically of exosomal MSN, played on the migration of normal endometrium *in vivo* and *in vitro* experiments. We observed that high expression of MSN in ectopic endometrial cells and exosomes was regulated by ERα recruiting E2 signaling to the RohA-Rock2-MSN pathway. It was intriguing to find that the knockdown of ERα did not affect the expression of Gα_13_, although a significant reduction was observed in downstream proteins expression. This might be explained by the fact that ERα and Gα_13_work synergistically in regulating this signaling pathway. However, further studies are required to get a full understanding underlying this phenomenon.

In this study, exosomal MSN could induce the normal endometrial cell migration and increase the number of the vascular lumen (lumen size did not change significantly vs. control group *in vitro* experiment). Meanwhile, the expression of inflammatory cytokines around the surrounding tissues of heterotopic foci was also up-regulated. Consequently, the adhesion of the ectopic region was increased, which is consistent with the results from previous studies (Bedir et al., 2016; Lee et al., 2020; Liu et al., 2020). The KEGG pathway and western blot analysis suggested that this biological phenomenon might be related to the up-regulation of MMP9 expression by MSN.

MMP9 is a member of the MMPs family, which is a protein family of zinc-dependent endopeptidases whose primary function is to degrade the extracellular matrix (ECM) and maintain the dynamic balance of the extracellular matrix. MMP9 involves tissue remodelling, organogenesis, inflammation, initiation of cancer, as well as other physiological and pathological processes (Bäck et al., 2010; Li and Wu, 2010). A number of studies have shown that the high expression of MMP9 is associated with the improvement of tumour migration and invasion ability (Guo et al., 2015; Li et al., 2015; Chen et al., 2018). In this study, the increased migration of normal endometrial cells was achieved through the up-regulated the expression of MMP9 mediated by exosomal MSN. Meanwhile, exosomal MSN might also promote angiogenesis and cellular inflammation by mediating the expression of MMP9. There is a study on the correlation between MSN and MMP9 showed that, in individual tumours, the expression of MSN and MMP9 remains consistent, which jointly promotes tumour metastasis (Lan et al., 2020). In our study, ectopic exosomal MSN promoted angiogenesis via MMP9/p-VEGFR signaling. Therefore, the migration and invasion of the ectopic cells are significantly weaker than that of the cancer cells. This suggests that endometriosis shares similar characteristics with tumours in certain aspects. However, it should be taken into consideration that the migration of normal endometrial cells is not the result of EMT, as it is mostly the case in cancer.

Not many studies provide evidence for the correlation between MSN and inflammatory factors. Nevertheless, it has been reported that the expression of MSN might be related to the recruitment of neutrophils. In the current study, the reduction in the expression of inflammatory factors caused by the knockdown of exosomal MSN might still be associated with the inhibition of MMP9 expression in cells. The relevant evidence will be verified continually in subsequent experiments. Besides, we found that exosomal moesin deficiency can suppress the size of the ectopic foci. However, in the *in vitro* experiment, ectopic exosomes did not promote the proliferation of normal endometrial cells (supplementary file1). Thus, we supposed that the growth of heterotopic foci was attributed to the migration of endothelial cells.

## Conclusion

In conclusion, ectopic endometrial cells derived exosomal MSN impacted the migration ability of eutopic endometrial cells via distal secretion manner. Meanwhile, we noticed that an abnormal estrogen concentration might partly contribute to the high expression of MSN in exosomes derived from ESCs. Furthermore, exosomal MSN induced the high expression of inflammatory cytokines in surrounding tissue of ectopic lesions, leading to the increase in cell adhesion and angiogenesis in surrounding ectopic tissue. Finally, the construction of a “migration-inflammation-vascularisation” loop connecting normal endometrium and ectopic endometrium facilitated the progression of endometriosis. Overall, our study suggested that exosome, with its important biological function as a carrier of genetic information between cells, could contribute to the development of EMS. Future exploration should be made further to understand the role of exosome in endometriosis.

## Data Availability

The original contributions presented in the study are publicly available. This data can be found here: URL: https://www.ebi.ac.uk/pride/ Accession No.: PXD033124.

## Supplementary Material

Supplementary Table S3 List of three up-regulated proteins and six down-regulated proteins based on fold change in exosomes from ESCs compare to NSCs. **(A)**: normal endometrial stromal cells (NSCs), **(B)**: Ectopic stromal cells (ESCs).supplementary-material.
