# Supplementary material for: Ectopic Endometrial Cell-Derived Exosomal Moesin Induces Eutopic Endometrial Cell Migration, Enhances Angiogenesis and Cytosolic Inflammation in Lesions Contributes to Endometriosis Progression
Source: Front Cell Dev Biol. 2022 Apr 26;10:824075. doi: 10.3389/fcell.2022.824075 (PMC9086167; doi:10.3389/fcell.2022.824075)
Supplement: Supplementary file 1 [file Table1.DOCX]

| Sample  Number | Gender | Age (y) | Pathological  Diagnosis | Menstrual  Cycle  (Days) | Ectopic Tissue Size  (mm) | Dysmenorrhea (Y/N) | | CA125 (U/ml) |
| --- | --- | --- | --- | --- | --- | --- | --- | --- |
| 1 | F | 34 | Ovaria  Chocolate cyst | 5/30 | 58 | | N | 64 |
| 2 | F | 27 | Ovaria  Chocolate cyst | 5/30 | 72 | | Y | 40 |
| 3 | F | 33 | Ovaria  Chocolate cyst | 5/29 | 64 | | N | 49 |
| 4 | F | 30 | Ovaria  Chocolate cyst | 6/31 | 55 | | Y | 51 |
| 5 | F | 32 | Ovaria  Chocolate cyst | 5/30 | 61 | | N | 45 |
| 6 | F | 33 | Ovaria  Chocolate cyst | 5/30 | 57 | | N | 47 |
| 7 | F | 27 | Abortion | 6/30 | - | | N | - |
| 8 | F | 38 | Myoma of uterus | 5/29 | - | | Y | 45 |
| 9 | F | 29 | Myoma of uterus | 6/31 | - | | N | 51 |
| 10 | F | 37 | Myoma of uterus | 5/30 | - | | N | 55 |
| 11 | F | 38 | Myoma of uterus | 5/29 | - | | N | 69 |
| 12 | F | 32 | Myoma of uterus | 5/30 | - | | Y | 46 |
| 13 | F | 29 | Abortion | 5/29 | - | | N | - |
| 14 | F | 30 | Abortion | 6/31 | - | | N | - |
| 15 | F | 35 | Myoma of uterus | 6/30 | - | | N | 75 |
| 16 | F | 36 | Myoma of uterus | 6/29 | - | | N | 55 |
|  |  |  |  |  |  | |  |  |
