# Supplementary figures and images for: Ectopic Endometrial Cell-Derived Exosomal Moesin Induces Eutopic Endometrial Cell Migration, Enhances Angiogenesis and Cytosolic Inflammation in Lesions Contributes to Endometriosis Progression
Source: Front Cell Dev Biol. 2022 Apr 26;10:824075. doi: 10.3389/fcell.2022.824075 (PMC9086167; doi:10.3389/fcell.2022.824075)

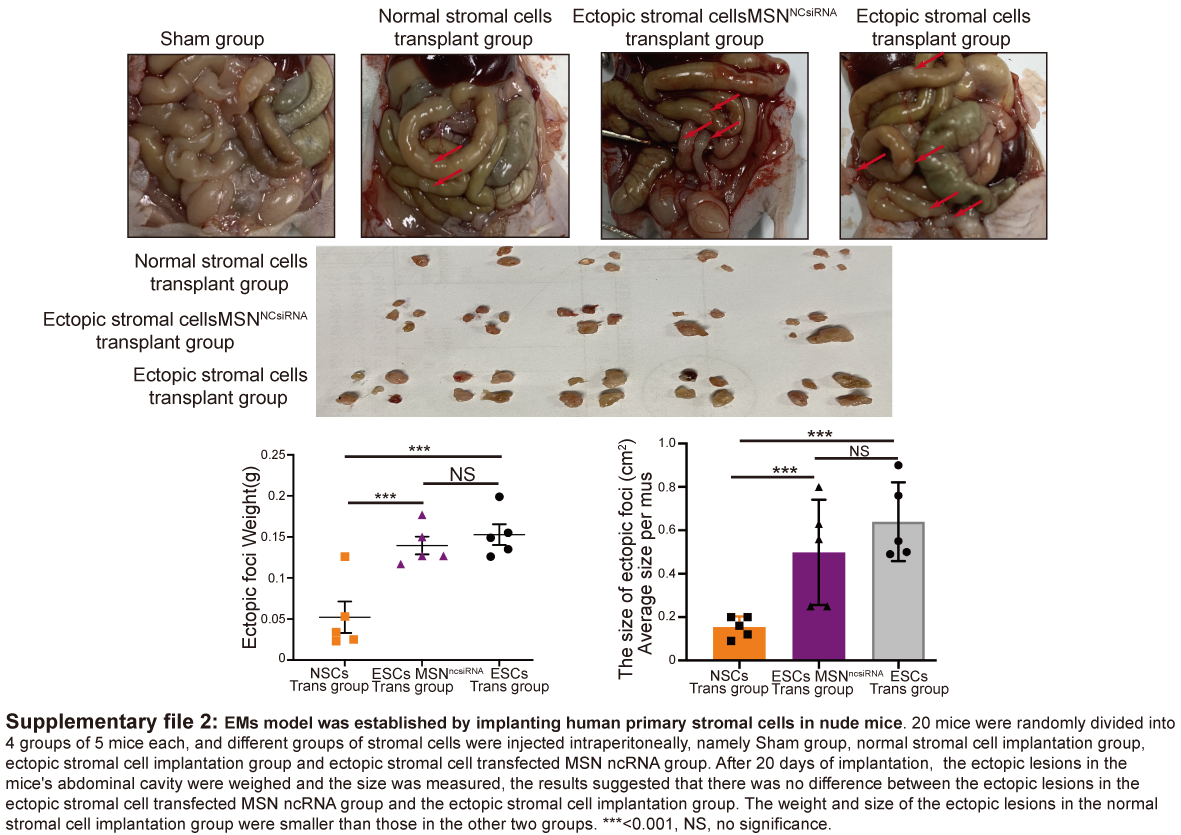

Supplement: Supplementary file 2 [file Image3.TIF]

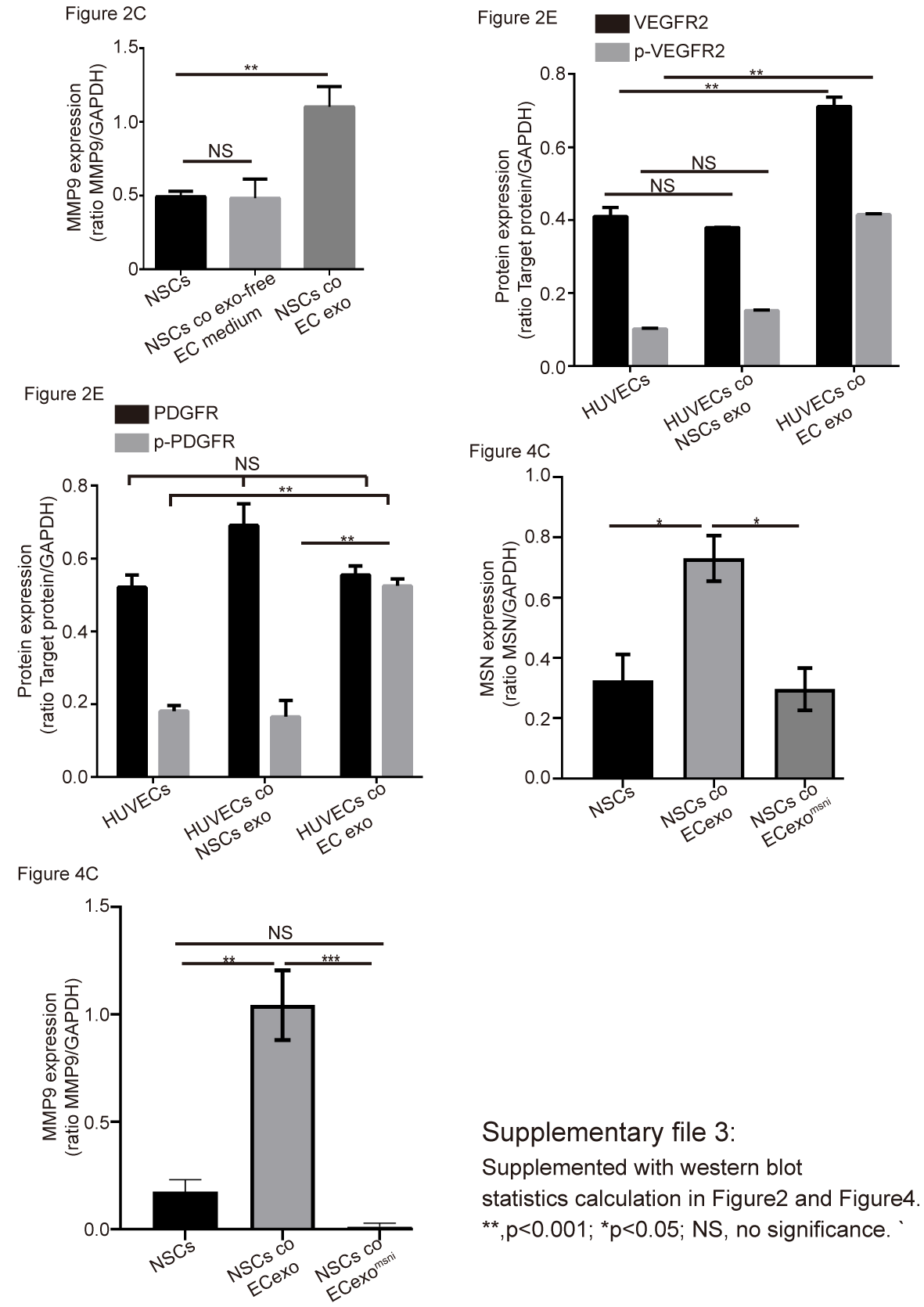

Supplement: Supplementary file 3 [file Image2.TIF]

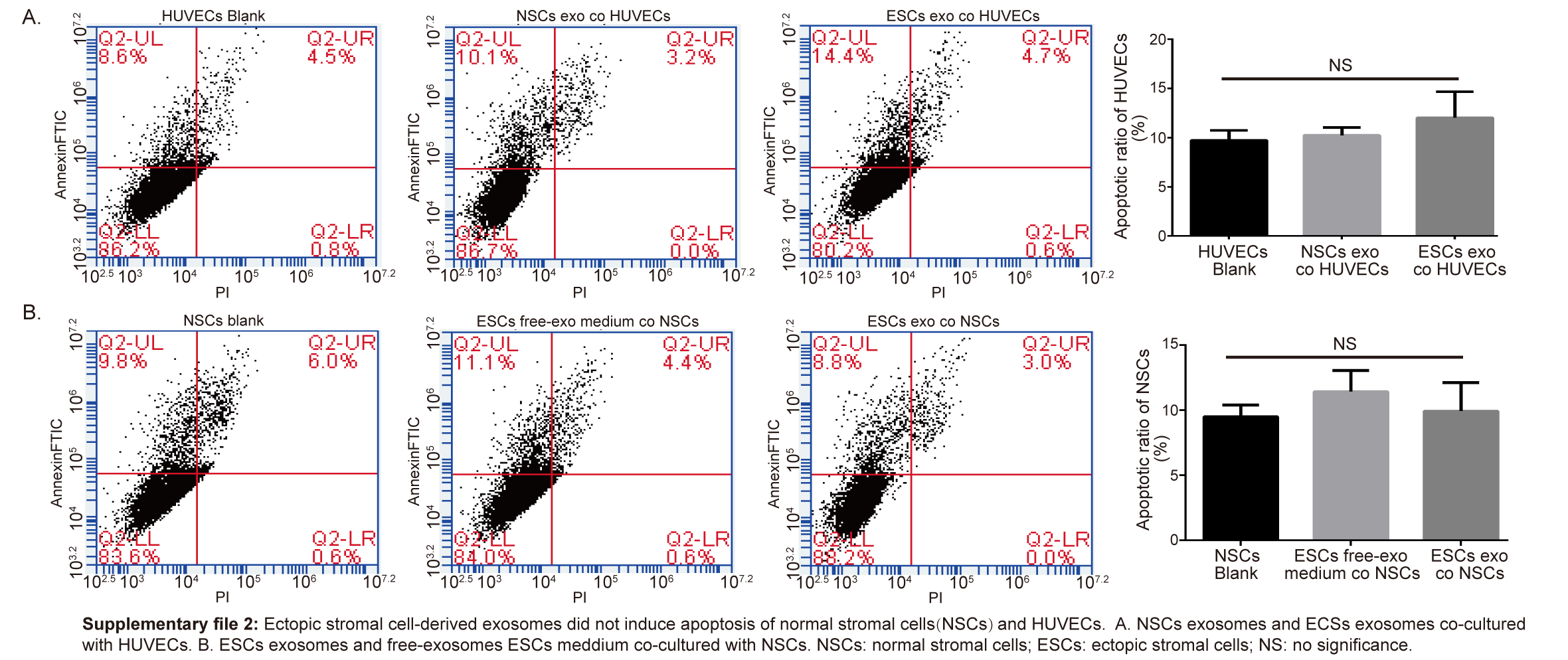

Supplement: Supplementary file 4 [file Image1.TIF]
