# Supplementary material for: Ectopic Endometrial Cell-Derived Exosomal Moesin Induces Eutopic Endometrial Cell Migration, Enhances Angiogenesis and Cytosolic Inflammation in Lesions Contributes to Endometriosis Progression
Source: Front Cell Dev Biol. 2022 Apr 26;10:824075. doi: 10.3389/fcell.2022.824075 (PMC9086167; doi:10.3389/fcell.2022.824075)
Supplement: Supplementary file 5 [file Table2.DOCX]

| Primer Name | Sequence 5’-3’ |
| --- | --- |
| hTNF-a F | CCTCTCTCTAATCAGCCCTCTG |
| hTNF-a R | GAGGACCTGGGAGTAGATGAG |
| hIL-1β F | ATGATGGCTTATTACAGTGGCAA |
| hIL-1β R | GTCGGAGATTCGTAGCTGGA |
| hIL-18 F | TCTTCATTGACCAAGGAAATCGG |
| hIL-18 R | TCCGGGGTGCATTATCTCTAC |
| hIFN-α1 F | GCCTCGCCCTTTGCTTTACT |
| hIFN-α1 R | CTGTGGGTCTCAGGGAGATCA |
| hICAM-1 F | ATGCCCAGACATCTGTGTCC |
| hICAM-1 R | GGGGTCTCTATGCCCAACAA |
| mTNF-a F | CCCTCACACTCAGATCATCTTCT |
| mTNF-a R | GCTACGACGTGGGCTACAG |
| mIL-1β F | GCAACTGTTCCTGAACTCAACT |
| mIL-1β R | ATCTTTTGGGGTCCGTCAACT |
| mIL-18 F | GACTCTTGCGTCAACTTCAAGG |
| mIL-18 R | CAGGCTGTCTTTTGTCAACGA |
| mICAM-1 F | GTGATGCTCAGGTATCCATCCA |
| mICAM-1 R | CACAGTTCTCAAAGCACAGCG |
