# Supplementary material for: Ectopic Endometrial Cell-Derived Exosomal Moesin Induces Eutopic Endometrial Cell Migration, Enhances Angiogenesis and Cytosolic Inflammation in Lesions Contributes to Endometriosis Progression
Source: Front Cell Dev Biol. 2022 Apr 26;10:824075. doi: 10.3389/fcell.2022.824075 (PMC9086167; doi:10.3389/fcell.2022.824075)
Supplement: Supplementary file 6 [file Table3.DOCX]

| **Protein _ID** | **Symbol** | **Entrez gene name** | **Sequence coverage [%]** | **Fold change(log2)（NE/EC）** | **P value (Significance )** |
| --- | --- | --- | --- | --- | --- |
| P62826 | RAN | GTP-binding | 23.6 | 2.93152 | 2.67E-05 |
| E9PRK8 | FTH1 | Ferritin | 31.6 | 2.06027 | 0.0043923 |
| J3QS39 | UBB | Ubiquitin-40S ribosomal | 36.6 | 2.03402 | 0.00511978 |
| A0A140TA49 | C4A | Complement C4-A | 5.8 | 1.87496 | 0.0128654 |
| A0A024QYT5 | SERPINE1 | Serpin peptidase inhibitor, clade E member 1 | 34.8 | 1.73553 | 0.0284192 |
| Q16853 | AOC3 | Membrane primary amine oxidase （AOC3） | 3.1 | 1.69569 | 0.0355097 |
| V9HWC0 | HEL70 | Moesin | 12.3 | 0.540539 | 0.010905 |
| A0A024R6R4 | MMP2 | Matrix metallopeptidase 2 | 34.1 | 0.515635 | 0.00600089 |
| V9HWD6 | HEL-S-1 | HUMAN Epididymis secretory protein Li 1 | 28.9 | 0.497566 | 0.00372578 |
